# Study on Various Chemical Systems for the Preparation and Application of Nickel Nanopastes for Joining Processes

**DOI:** 10.3390/ma18071411

**Published:** 2025-03-22

**Authors:** Benjamin Sattler, Susann Hausner, Guntram Wagner

**Affiliations:** Group of Composites and Material Compounds, Chemnitz University of Technology, 09125 Chemnitz, Germany; susann.hausner@mb.tu-chemnitz.de (S.H.); guntram.wagner@mb.tu-chemnitz.de (G.W.)

**Keywords:** nanojoining, nanoparticle, nanopaste, paste formulation, sintering, nickel, alternative to brazing, low-temperature joining, energy efficiency, mild steel joining

## Abstract

Nanojoining, which utilizes nanoparticles for joining applications, is an interesting method that stands out from conventional processes by combining relatively low joining temperatures with high service temperatures. To use the nanoparticles for this purpose, it has proven useful to process them as a paste. The chemical composition of such a nanopaste has a certain influence on the properties ultimately achieved by the joint. While nickel nanoparticles represent the metal content of the here investigated nanopastes, a variety of substances can be utilized as organic components to form the actual paste-like suspension. Derived from the literature on nanoparticle synthesis, a variety of candidates were identified from which numerous paste compositions were developed for this work. So, high metal content (70 wt.%) nickel nanopastes were prepared from these solvent–stabilizer systems by ultrasound-enhanced mixing. The study evaluates the pastes in terms of manufacturability and handleability. The findings reveal insights into the effects of different chemical substances. Additionally, joining tests using the mild steel DC01 are presented, demonstrating the impact of the paste composition on the joining strength and the microstructure of the joint as well. Within this study, a paste consisting of terpineol and KD4 was the most favorable.

## 1. Introduction

It is widely recognized that materials at the nanometer scale exhibit lower melting and sintering temperatures due to their increased surface-to-volume ratio and increased specific surface energy [1,2,3]. This effect enables a joining technology at comparatively low processing temperatures (nanojoining) by using metallic nanoparticles. During the joining process the nanoparticles sinter and coalesce, creating a joint seam material with almost the same melting temperature as the corresponding bulk material [4,5]. This characteristic facilitates both low joining temperatures and high service temperatures at the same time. The first materials used for this purpose were silver and copper [4,6,7,8] for, e.g., applications in electronic packaging. The nanoparticles are preferably used in the form of a paste (nanopastes), with the metallic particles representing the main component. So, from a physical point of view, nanopastes are mixtures, more precisely suspensions, in which nanoparticles (at high content) are dispersed in a liquid phase. The liquid phase can consist of one or more substances which act as solvents and stabilizers (referred to here as organics or organic content/components). It is obvious that the organic content influences how a paste behaves in terms of handling but also in the joining process. So ultimately the joining result depends on the choice of organic components. There are already numerous studies on compositions of Ag and Cu nanopastes [9,10,11]. Thus, such products can already be purchased and used in industry. Driven by applications that typically depend on high-temperature brazing such as industrial furnaces and gas turbines [12,13,14], nanojoining with nickel nanoparticles has recently been investigated for joints subjected to higher mechanical loads and elevated operating temperatures [15,16,17]. While these studies confirm the feasibility of a nanojoining process using Ni nanoparticles, hardly any research has focused on the composition of nickel nanopastes. To the best of our knowledge, such pastes are also not commercially available. Since the interactions between the nanoparticles and the paste organics are complex, the impact on the joining result is difficult to predict and preferably substances are not trivial to select. This needs to be investigated through experimental testing. Paste formulation must further be compatible with the joining temperatures used for nickel nanoparticles, which are significantly higher than those of Ag or Cu nanoparticles, due to the higher melting point of nickel. The joining parameters used here are explained in more detail in Section 2.4.

Overall, there is a potential for research, so this study aims to contribute to the understanding of how the chemical composition of a nickel nanopaste influences its properties and the joining seam formation.

## 2. Materials and Methods

### 2.1. Nanoparticles, Selection of Chemical Components for Paste Preparation and Base Material for Joining Samples

**Figure 1:**
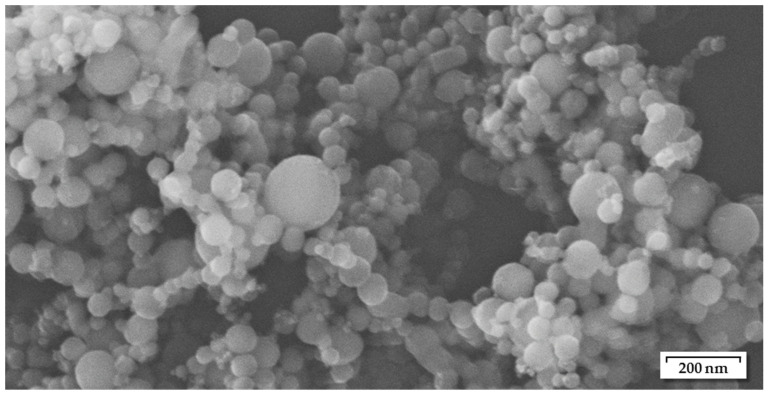
Nickel nanoparticles in as-received condition, used for the preparation of Ni nanopastes, mean diameter 60 to 80 nm according to the manufacturer.

For this investigation, nickel nanoparticles (Ni-NP) with 99.7% purity and diameters of 60–80 nm were used, purchased from Skyspring Nanomaterials Inc. (Houston, TX, USA). The specifications are provided by the manufacturer, whereas the particle size range could generally be confirmed by SEM imaging (Figure 1).

Since the aim of this study is to investigate the influence of the chemical components of a nanopaste, a comprehensive literature research was carried out to identify candidates that can act as solvents or stabilizers in a (nickel) nanoparticle suspension. Table 1 contains all found candidates, the associated references (mostly papers on NP synthesis), and further information, such as aspects related to human health. An important health-related aspect is that during handling (pre-drying) and in the joining process itself, the organics of a nanopaste evaporate/decompose due to heating (intentionally). This can lead to a certain level of exposure for the personnel involved, even with an air extraction system in place. Therefore, more harmless substances were preferred over hazardous ones and so the down selection led finally to 12 candidates which were used for this work (highlighted by an underlined “included”).

The base material for all joining samples is a 3 mm sheet of mild steel DC01 (material number 1.0330). Joining samples were prepared as described in Section 2.3 and Section 2.4 and were used for mechanical testing to determine joint shear strength and investigate its microstructure, respectively.

**Table 1:** List of candidates for substances that can act as solvents and stabilizers in a nickel nanopaste.

Solv./Stab. ^(1)^	Chemical Compound|Investigated in	Cond. at RT ^(2)^	CAS No.	Remarks
solv.	Dihydroterpineol|[18]	L	498-81-7	no advantage over Terpineol ^(3)^
	Dimethylformamide (DMF)|[19]	L	68-12-2	not used, hazardous to health [20]
	Dioctyl ether|[21]	L	629-82-3	included
	Ethylen glycol|[22,23]	L	107-21-1	not used, hazardous to health [24]
	Hydrazine|[25]	L	302-01-2	not used, highly toxic [26]
	Oleylamine|[27,28,29]	L	112-90-3	not used, hazardous to health [30]
	Paraffin (paraffin wax)|[31,32,33]	L	8012-95-1	included
	Polyethylenimine|[34]	L	9002-98-6	not used, hazardous to health [35]
	Phenol ether/Phenyl ether|[27,36]	L	(various)	not used, hazardous to health [37,38]
	Terpineol|[17,39,40]	L	8000-41-7	included
	Tetrahydrofuran (THF)|[41]	L	109-99-9	not used, hazardous to health [42]
	Toluene|[21,43]	L	108-88-3	not used, hazardous to health [44]
	Trioctylamine|[36]	L	1116-76-3	not used, hazardous to health [45]
	Water|[19,25,46,47,48]	L	7732-18-5	included
both	Oleic acid|[34,43,49,50,51] and more	L	112-80-1	included
	PEG 400 ^(4)^|[17,23,52]	L	25322-68-3	included
stab.	CTAB ^(5)^|[47,53]	S	57-09-0	included
	Glycolic acid|[54,55]	S	79-14-1	included
	HYPERMER™ KD4 (now: LP1)|[17,39]	L	(none)	included
	Lauric acid, Palmitic acid|[36,56]	S	(various)	not used here in favor of Stearic acid ^(6)^
	other commercial surfactants|[18]	L	(none)	not used here in favor of KD4 ^(3)^
	Polyvinylpyrrolidone (PVP)|[22,25]	S	9003-39-8	included
	Sodium dodecyl sulfate (SDS)|[19,48,52]	S	151-21-3	included
	Stearic acid|[44,57]	S	57-11-4	included
	Urea|[58,59]	S	57-13-6	only employed in DES ^(7)^
	Xanthate|[60,61]	S	(various)	not used, hazardous to health [62]

^(1)^ Role of the chemical substance, so solvent or stabilizer (or both). ^(2)^ Condition of substance, so liquid or solid at room temperature. ^(3)^ Investigated in our own previous studies [17,63]. ^(4)^ Polyethylene glycol, “400” refers to the average molecular weight (g/mol). ^(5)^ Cetyltrimethylammonium bromide. ^(6)^ Stearic acid (saturated fatty acid) allows a direct comparison to oleic acid (unsaturated f. a.) due to its identical carbon chain length. ^(7)^ Deep eutectic solvents, a certain kind of ionic liquids.

### 2.2. Preparation of Ni Nanopastes

For producing the nanopastes, Ni-NP, and the organic substances were precisely weighed by adding each ingredient sequentially into a sample vial. The metal content for all pastes was set to 70 wt.% since this value was found to be close to the maximum for reliably preparing a paste with Ni-NP in this size range [63]. To achieve homogeneous mixing, ultrasound was used (device: Hielscher-Ultraschall-Technologie USP 200S, Teltow, Germany). The ingredients in the vial were exposed directly to the sonotrode (24 kHz, pulsed) for a one-minute duration, followed by manual stirring with a spatula. This cycle was repeated five times for a single mixture. The temperature was maintained at approximately room temperature by a water bath surrounding the vial. According to this method, 2.0 g nanopaste was obtained in a batch.

For determining useful nanopaste compositions for this study, it must be recognized that the literature references from which the candidates were taken (Table 1) almost exclusively describe processes for nanoparticle synthesis. This is important because for once, the metal content of the nanopastes to be produced here is much higher (70 wt.% is intended for all pastes) than suspensions for particle synthesis (approx. 1 wt.%). Secondly, the pastes here utilize already existing NPs, whereas NPs first emerge from crystallization in a synthesis. So, in order to adopt the findings from the literature, certain aspects such as the miscibility of different components or paste handling properties (i.e., spreadability) must be verified experimentally to develop suitable compositions. This is carried out in Section 3.1, divided into the type of solvent (water-based or organic). Finally, 13 different paste compositions could be used for joining tests. The composition of these, with a particular focus on stabilizer content, is discussed in detail in Section 4.

### 2.3. Nanopaste Application

Before nanopaste was applied to the steel samples, wet grinding was carried out to finish the surfaces using a machine with a rotating 600-grit SiC pad. This was performed while maintaining the sample parts in a fixed alignment, tangential to the grinding wheel. After finishing, the surfaces are cleaned (ethanol rinsing after ultrasonic bath) and dried. Then, a small amount of nanopaste was evenly spread on every part with a spatula and, afterwards, dragged beneath a peel bar with a 50 µm gap to set the paste layer to a defined thickness. It is important to note that this paste application of 100 µm in total, coming from two sample parts forming one joint, does not represent the thickness of the final joining seam. Due to the evaporation of the organics and the consolidation of the NP, the seam in the final joint is reduced to around 25 µm.

After application, the paste was pre-dried by heating the sample parts on a hot plate at a certain temperature and holding time. These parameters need to be adjusted depending on the composition of the respective paste. Proper pre-drying takes place between the limits of insufficient evaporation of the organics (paste still “wet”) and excessive heating, which can lead to unintended early sintering of the nanoparticles or oxidation effects that are detrimental to the actual joining. Therefore, (near) optimum parameters were determined here in preliminary tests with regard to achievable shear strength for each solvent system used for paste preparation. The found values are listed in Table 2 whereby the parameters for terpineol and PEG-based pastes were already figured out in a prior investigation [63].

### 2.4. Joining Sample Geometry, Joining Process and Parameters

**Figure 2:**
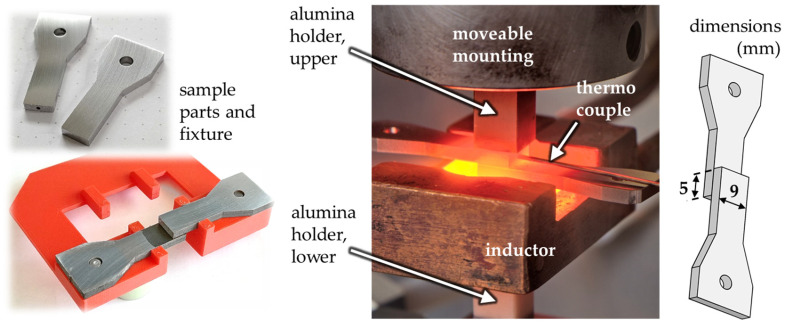
Sample parts and the fixture at the left, heated sample during joining process at center, joined sample at the right.

Two sample parts with pre-dried nanopaste were placed in a special fixture which ensures an overlapping section of 5 mm between the two parts, which are 9 mm wide. This is illustrated in Figure 2. The sample arrangement in its fixed position is then introduced into the chamber of the actual joining device, where a pair of alumina holders clamp the overlapping sample parts so that the fixture can be removed. The upper holder is movable, which allows a joining pressure to be applied on the overlap area of the sample by an external force. The joining process takes place in a high vacuum at approx. 4 × 10^−4^ mbar or lower to avoid oxidation. A thermocouple is attached to a drill hole in one of the sample parts, by which the temperature-time sequences can be controlled during the joining process. The components of the joining device where the sample arrangement is inserted are shown also in Figure 2. Every joining sample is heated inductively at a rate of 150 K/min until 675 °C is reached. This temperature was maintained for 2 min followed by free cooling. During the whole process, a joining pressure of 9.6 MPa was applied via the alumina holders. The parameter set was chosen so that the samples are joined at “critical” (relatively low) values for temperature, holding time, and joining pressure, with reference to Ni-NP. Previous studies have identified approximately 675 °C as the minimum temperature at which Ni-NP can form usable bonds [17]. The so-produced joints do not achieve the highest strengths, but react more sensitively to the different paste compositions and thus enable a good differentiation of results. In addition, the potential of nanojoining lies at low process temperatures, meaning that results for this range are of interest.

### 2.5. Shear Strength Testing and Microstructure Analysis

The joining samples represent an overlap configuration (see Figure 2). The shear strength of the joints is measured by using a testing machine (Zwick Allround-Line, 20 kN, Ulm, Germany) with an integrated vise that compensates for the vertical offset caused by the overlapping geometry. The clamped samples were loaded with an initial force of 100 N before a quasi-static shear rate of 10^−3^/s (0.005 mm/s) was applied. Testing was continued until sample failure to determine the maximum shear strength.

Additional joining samples were subjected to metallographic preparation to analyze their microstructure by SEM imaging using Zeiss LEO 1455VP and Zeiss NEON 40EsB (Oberkochen, Germany).

## 3. Results

### 3.1. Evaluation of the Manufacturability of Different Nanopaste Compositions

#### 3.1.1. Water-Based Nanopastes

**Figure 3:**
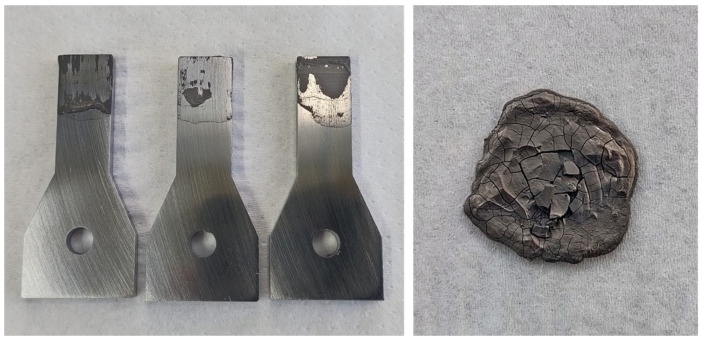
(**Left**): quickly dried spots prevent uniform paste application. (**Right**): paste dries out completely after several days.

The first composition to be tested is a simple mixture of nanoparticles in distilled water. Dispersing the Ni-NP in distilled water using ultrasound initially works well and the resulting paste is easy to spread with a spatula. However, problems arise in further handling. When applied as a thin layer to a sample surface, the paste dries within seconds. So, peeling off to a uniform 50 µm paste layer as intended is not possible due to the already dried spots (see Figure 3) which were left. In addition, the paste has a very limited shelf life. Even when stored at 8 °C in a closed container, the paste dries out within a few days and becomes unusable (see Figure 3, right).

In order to still estimate the potential of these purely water-based Ni nanopastes for the joining process, two joining samples were prepared by manual nanopaste application using a spatula and joined under the reference parameters. However, both joining samples broke already at little force by hand, so in contrast to organic paste compositions examined later, no usable strength was shown.

**Figure 4:**
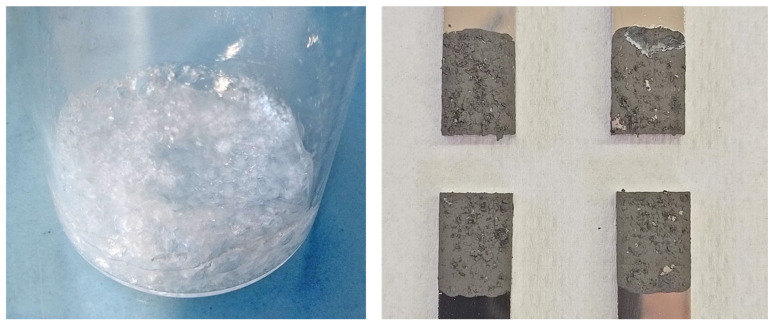
Crystallized CTAB pre-solution (which also affects the paste) and unfavorable paste application properties on the joining sample surface.

The next approach is adding a stabilizer to an aqueous paste. The first candidate is the (solid) CTAB, which was prepared in a 20% aqueous pre-solution, which had to be heated therefore. For the actual paste, distilled water and a certain amount of the CTAB pre-solution were then added to the Ni-NP. However, with a metal content of 70 wt.% a pasty mixture was not possible, so more pre-solution had to be added. The final composition was Ni-NP 46.1/H_2_O 47.8/CTAB 6.1 (by wt.%). For the resulting paste, the water continues to evaporate very quickly. Another negative aspect is that CTAB crystallizes from the paste, which could also be observed in the pre-solution (Figure 4, left) which prevents an even layer of paste (Figure 4, right). Another approach using SDS (at a content of 8.4 wt.%) as a stabilizer instead of CTAB led to no improvement in terms of very quick dry-up.

It can be stated that water, even with the addition of stabilizers, does not allow the formation of a usable Ni nanopaste for the purpose of joining as intended in this work and is therefore not further considered.

#### 3.1.2. Nanopastes from Organic Solvents

A larger and more promising field is provided by organic solvents. Based on the findings of Table 1 and our own preliminary work [17], a variety of combinations of the candidates for solvents and stabilizers was experimentally evaluated for the miscibility of stabilizers (partially solid substances). The results are shown in Table 3 and explained in more detail below.

*Solvent Dioctyl Ether (DiOE) with Fatty Acids*

**Figure 5:**
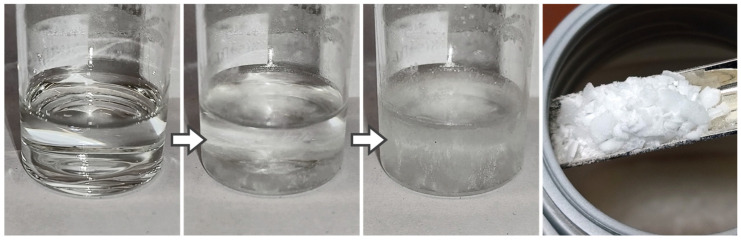
Precipitating stearic acid in dioctyl ether upon cooling (arrow indicates timeflow); **right:** pure stearic acid.

Oleic acid (OA) and stearic acid (SA) are fatty acids which only differ in their eponymous hydrogen saturation and their molecular shape. Considering both representatives enables a direct comparison of the stabilizer effect of saturated (stearic acid) and unsaturated fatty acids (oleic acid). As shown in Table 3, OA is easily soluble in DiOE whereas a mixture with (solid) SA needs to be heated to around 80 °C. Figure 5 shows the separation of SA when the mixture with DiOE cools down.

For nanopaste preparation, equal amounts of OA and SA are used for the respective compositions to ensure comparability. The content fractions used here and for all other pastes are discussed in detail in Section 4. The resulting paste compositions are as follows:

- Ni (70%)_DiOE_OA (15%);
- Ni (70%)_DiOE_OA (1.5%);
- Ni (70%)_DiOE_SA (15%);
- Ni (70%)_DiOE_SA (1.5%).

(refers to wt.%; “Ni” means Ni-NP; solvent is always balanced).

**Figure 6:**
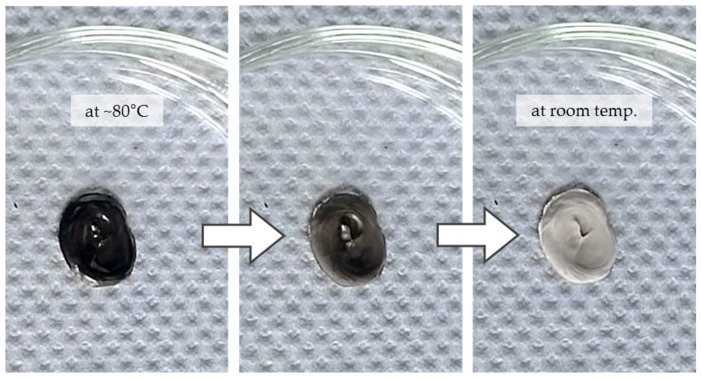
Behavior of stearic acid in a nanopaste; precipitates are formed when cooled to room temperature.

The SA-containing pastes were difficult to prepare and handle because a temperature of around 80 °C must be sustained to keep them liquid. As soon as the paste cools down, the stearic acid solidifies in a very fine distribution causing white precipitates which turn the paste into a brittle mass (see Figure 6). Although this strongly limits the usability of the paste, the white coloration represents an optical indicator for the (very homogeneous) distribution of the stabilizer within the paste, which might be of interest in other areas, such as for analytical purposes.

**Figure 7:**
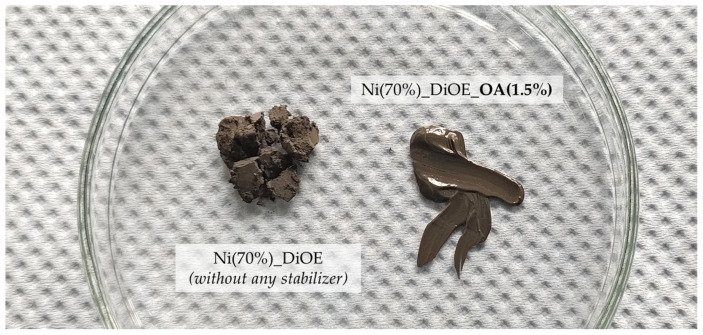
Left: mixture of 70 wt.% Ni-NP and dioctyl ether; right: same paste composition, but 1.5 wt.% oleic acid as stabilizer added.

For reference purposes, an attempt was also made to prepare a stabilizer-free DiOE paste. Surprisingly, it turns out that this mixture cannot be processed into a paste at all, although the metal content was still at 70 wt.% (as for all others). Even after intensive ultrasound-assisted dispersion, no suspension was achieved, only a moist, crumbly mixture (see Figure 7). The entire process was repeated again with new ingredients in order to rule out experimental errors. The result was identical. Thus, the stabilizer component had a decisive influence on the manufacturability, at least with dioctyl ether as the solvent.

*Solvent Paraffin with Fatty Acids*

This solvent–stabilizer system corresponds to the one described above, but with paraffin as the solvent. The paraffin used here has a (kinematic) viscosity of 52 mm^2^/s at 40 °C and a boiling point of 218 °C. It is liquid at room temperature. The same stabilizer contents are used as for the DiOE-based pastes, but according to the previous findings, only oleic acid was considered (since stearic acid also precipitates in a paraffin solution):

- Ni (70%)_Pf_OA (15%);
- Ni (70%)_Pf_OA (1.5%).

Here, again, a stabilizer-free nickel nanopaste made from pure paraffin, so Ni (70%)_Pf is not possible. These are surprising results since stabilizer-free pastes with a metal content of 70 wt.% can be produced using other investigated solvents like water (see Section 3.1.1), terpineol, or PEG 400 (both follow down below).

*Glycolic Acid (GA) as Stabilizer in Suitable Solvents (Terpineol, PEG)*

The solubility of GA in various organic solvents had to be evaluated first since the literature only describes aqueous solutions. The results show that GA can only be used in combination with terpineol (in low concentration) and PEG 400, as presented in Table 3. However, during the preparation of the composition Ni (70%)_PEG_GA (15%) it became apparent that the mixture was not able to form a paste. Accordingly, only pastes with low stabilizer concentration (1.5 wt.% GA) could be realized:

- Ni (70%)_T_GA (1.5%);
- Ni (70%)_PEG_GA (1.5%).

*Polyvinylpyrrolidon (PVP) and KD4 as Stabilizer in Suitable Solvents (Terpineol, PEG)*

**Figure 8:**
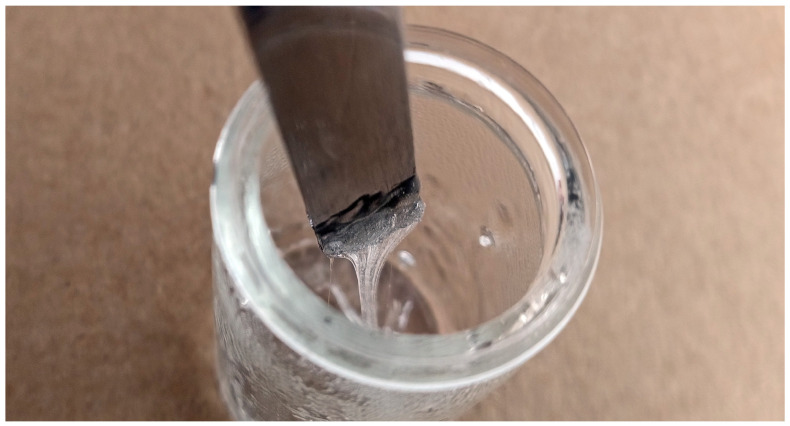
Pre-solution made from 200 mg PVP and 400 mg terpineol after cooling down to room temperature, resulting in an almost unprocessable adhesive-like mixture.

As for GA, it turned out PVP can also only be used for pastes based on terpineol and PEG 400. However, if concentrations in the range of 1:2 (PVP to solvent, by weight) are tested, the viscosity of the mixture becomes very high, up to adhesive-like values, especially for PVP in combination with terpineol (see Figure 8). This would correspond to a stabilizer content of 10 wt.% in the paste.

Taking these limitations into account, the following pastes are prepared using PVP, including some stabilizer-free reference compositions as well as the combination of terpineol and KD4:

- Ni (70%)_T_PVP (1.5%);
- Ni (70%)_T_KD4 (0.7%) (composition already used in previous studies);
- Ni (70%)_T (stabilizer-free T-based nanopaste);
- Ni (70%)_PEG_PVP (1.5%);
- Ni (70%)_PEG (stabilizer-free PEG 400-based nanopaste).

### 3.2. Evaluation of the Shear Strength of Joining Samples

By using all the paste compositions presented and selected in Section 3.1, joining samples are produced according to the procedures described in Section 2. Mechanical testing is then carried out on 2 repeating samples to determine the achievable shear strength achieved by every nanopaste composition. The achieved shear strengths are summarized in Table 4.

It should be noted that these shear strength tests based on two repeating samples do not provide a high degree of statistical validation and are instead intended as exploratory experiments to identify promising paste compositions. After evaluating the results, they reveal distinct trends that provide meaningful insights, which are presented below.

The series of pastes based on DiOE (dioctyl ether) were tested with OA (oleic acid) and SA (stearic acid), so two different fatty acids. A high OA content leads to a higher shear tensile strength of 70 MPa. With a 10 times reduced content of OA, 52.9 MPa can be achieved. For the SA-stabilized pastes, the values are consistently lower. Both stabilizer contents of 15 and 1.5 wt.% lead to around 30 MPa.

For paraffin-based pastes, the higher content of OA also leads to higher shear tensile strength. In a direct comparison, however, the value achieved is lower than the corresponding DiOE-based pastes.

Pastes based on terpineol lead to the best results here. A paste containing terpineol can also be produced without a stabilizer with 70 wt.% metal content, but this does not lead to reliable bond formation. One of the two joining samples broke when clamped into the testing machine, which is also reflected in the microstructure. When a stabilizer is added, glycolic acid, PVP, and finally KD4 lead to the highest shear strength in ascending order. The latter composition achieves the best joining result of all at 98.5 MPa with very little scattering.

**Figure 9:**
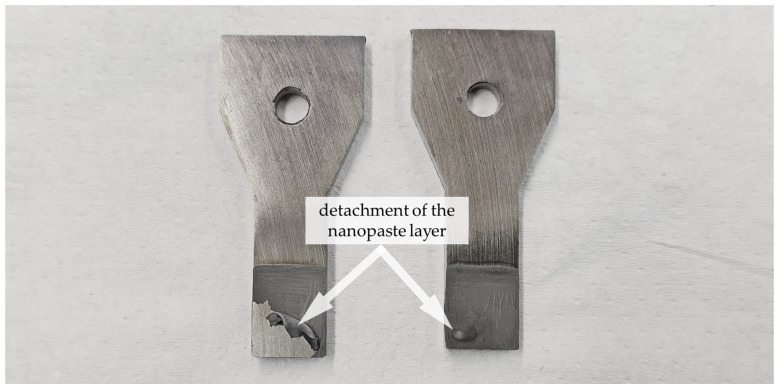
Detachment of the pre-dried paste layer in the case of two sample parts with paste Ni (70%)_PEG_GA (1.5%).

Within the series of nanopastes containing PEG 400, it is possible to work without a stabilizer. Adding 1.5 wt.% PVP actually worsens the joining result; with 1.5 wt.% of glycolic acid, approximately the same strength is achieved with 52 MPa. While preparing the samples, a partial detachment of the paste layer after pre-drying was observed in the case of pure PEG paste and the variant with GA (see Figure 9). This occurred in about half of all individual sample parts, so a reliable paste application was not given.

### 3.3. Evaluation of the Microstructure of the Joints

To support results from the mechanical testing, the microstructure of the joint is examined. It should be noted that the formation of a joining seam during nanojoining is subject to a certain degree of scatter, especially at low joining parameters. For example, at the beginning of the joining process, some areas sinter more quickly and thus receive more load, while a weaker bonding exists in other places. Images here are chosen to represent characteristics of the entire joining seam as best as possible.

**Figure 10:**
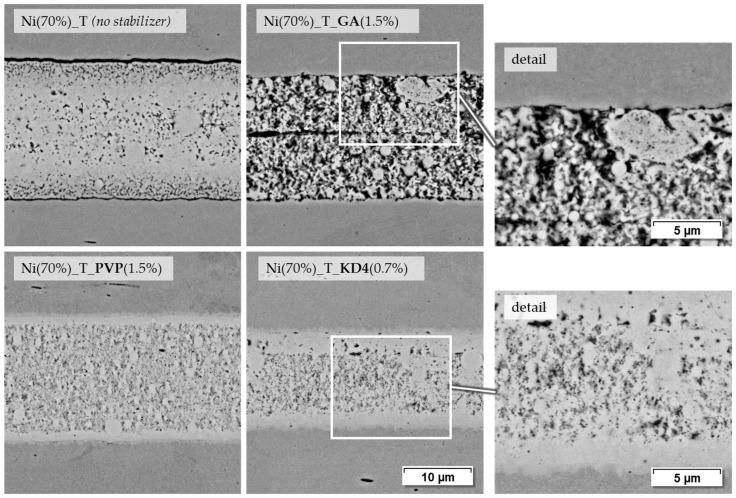
Joining zones of DC01 with terpineol-based nickel nanopastes, joined at 675 °C for 120 s and 9.6 MPa joining pressure (2000× and 4000× BSD images by SEM).

The pastes based on terpineol and PEG are well suited to demonstrate the influence of stabilizers, as they can also be produced without stabilizers. Figure 10 shows a series of SEM images of the terpineol-based pastes with more magnification on some details.

Paste Ni (70%)_T, the pure mixture of Ni-NP and terpineol, forms a relatively dense joint, which, however, shows hardly any bond to the base material. This is also supported by the highly scattering shear test strengths of the corresponding samples. A reproducible joining process is hardly possible in this case. Adding GA, as represented by paste Ni (70%)_T_GA (1.5%), results in a higher porosity in the joint and probably more pyrolytic residue from the paste organics. In the center of the joint, the separation of the two originally applied paste layers is still partially visible. However, some contact points are visible at the interface to the base material. The PVP containing paste Ni (70%)_T_PVP (1.5%) leads to a denser joint with at least visually good bonding to the base material. This also correlates with the strength values, which are higher than the paste containing GA. The paste with KD4 as a stabilizer also shows a relatively dense joint with good base material bonding, as can be seen in the magnified section. Despite the similar microstructure, the strength of these samples is even higher at 98.5 MPa than in the case of the PVP-containing paste at 57.4 MPa. This shows that the density or porosity of a joint only allows limited statements to be made about the mechanical durability of the sintered particles among themselves or to the base material.

**Figure 11:**
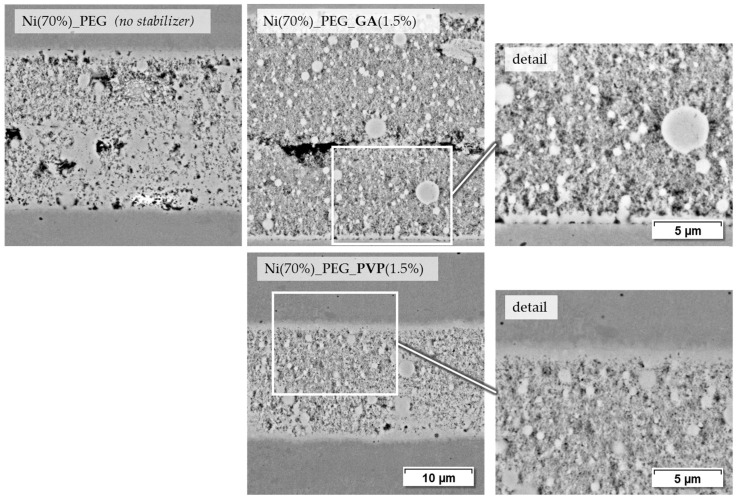
Joining zones of DC01 with PEG-based nickel nanopastes, joined at 675 °C for 120 s and 9.6 MPa joining pressure (2000× and 4000× BSD images by SEM).

Another series of joints is shown in Figure 11. Paste Ni (70%)_PEG, which consists only of Ni-NP and PEG, leads to mechanically durable joints and shows a relatively dense joint seam with partial base material bonding. When GA is added as a stabilizer, the seam appears somewhat less dense and in some places a slight separation between the paste layers applied on both sides is visible. There are contact points at the interface to the base material. The corresponding shear test samples are on the same level in terms of strength as those with stabilizer-free paste. PVP as a stabilizer, as with the terpineol-based pastes, leads to at least a visually good bond to the base material and a relatively dense joint seam formation. However, as already mentioned, the corresponding joint samples do not have any greater strength than the stabilizer-free PEG paste.

**Figure 12:**
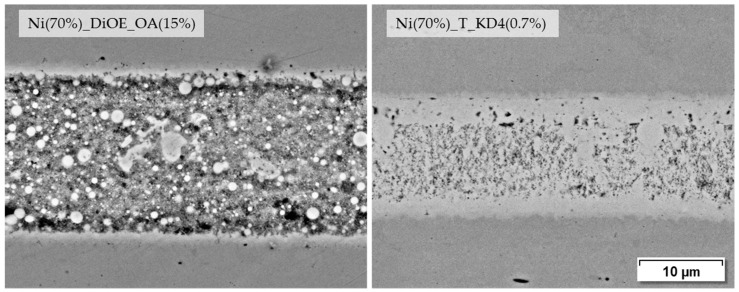
Joining zones of DC01 with dioctyl ether and terpineol-based pastes, joined at 675 °C for 120 s and 9.6 MPa joining pressure (2000× BSD images by SEM).

Among the DiOE-based pastes, the composition with the highest shear tensile strength Ni (70%)_DiOE_OA (15%) shows a uniform, but in comparison to others, less dense microstructure with visible base material bonding. In terms of shear tensile strength, only the previously described paste Ni (70%)_T_KD4 (0.7%) achieves an even higher value at 98.5 MPa. Figure 12 shows the joint seams of both pastes in direct comparison.

**Figure 13:**
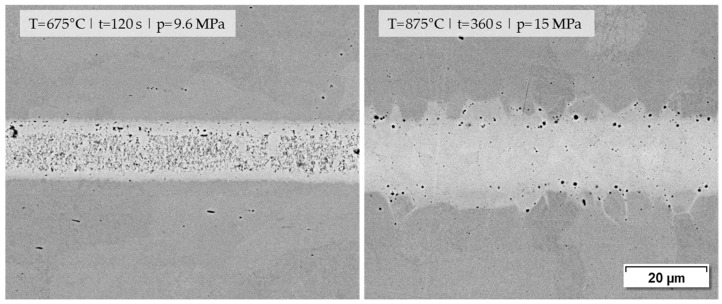
Joining zones of DC01 with nanopaste Ni (70%)_T_KD4 (0.7%) at different joining parameters for (T) temperature|(t) holding time|(*p*) joining pressure (1000× BSD images by SEM).

Concerning the microstructure images and the joining strengths achieved, it should be emphasized that low joining parameters were used in this investigation on purpose in order to obtain results that can be differentiated well. The microstructure of such a joint formed by a nanopaste can be significantly improved when the joining parameters are increased slightly, as shown in Figure 13. The joining sample on the right achieved a shear strength of 189.7 MPa, whereas the sample on the left reached 98.5 MPa.

## 4. Discussion

An important finding of this study was that water is not a suitable base for preparing nickel nanopastes with a high metal content. Therefore, the work is focusing on the investigation of organic substances. As a side note, first screening tests of relatively high volatile organic substances such as ethanol and isopropanol showed the same difficulties as water, so they do not also lead to useful pastes. These were, therefore, excluded from the study at the beginning.

The identification of useful candidates was achieved by literature research, which is covered in Table 1. For producing own nanopastes, the content fraction of the stabilizer is an important aspect. So, in the following, the composition of the nanopastes produced here is discussed. In theory, a reasonable stabilizer content is related to the specific surface area of the particles in the suspension (and thus to the particle size). Under certain assumptions (equally sized particles, single molecular layer stabilization, full efficiency) there are geometry-based calculation models for this [64]. However, on the one hand, this does not reflect real-world conditions and on the other hand system characteristics are required that are not readily available, such as the effective diameter of the functional polar group. To still obtain values for stabilizer contents, numerous references were evaluated for how much stabilizer mass was used in relation to the mass of (nickel) nanoparticles. The sources indicate that very different contents are used in practice. For example, Pascu et al. [28] obtained Ni-NP with a diameter of about 15 nm in the synthesis using 560.7 mg OA to 59 mg Ni (calculated from the mass of the metal salt). This corresponds to a stabilizer-to-nickel ratio of 9.55. In comparison, a synthesis by Zacharaki et al. [43] was carried out with 35.6 mg OA (40 µL) to a mass of 85 mg Ni, particles in the range of 5 nm emerged from. The stabilizer-to-nickel ratio amounts to 0.42 in this case. This means that Pascu et al. used about 22 times more OA as a stabilizer than Zacharaki et al., although the generated particles are even larger and therefore have less specific surface area. If all researched sources are considered, the range of stabilizer-to-metal mass ratios extends from 0.23 [25] to 16.75 [55]. Of course, different particle sizes were synthesized in these references, but no specific correlation between stabilizer content and the specific surface area of the particles can be derived. This points to a wide usable range of stabilizer content fractions and that supersaturation of stabilizer is acceptable.

For a nanopaste with 70 wt.% metal content as used here, the theoretical limit for the stabilizer-to-nickel ratio is 0.3 ÷ 0.7, so 0.43 (by mass). This would mean that the entire organic part of the paste consists only of stabilizer. Since many stabilizers are solids, they must of course be dissolved in a solvent, which represents the second organic component. As the miscibility tests of this study show (Table 3), a 1:1 ratio by weight is possible for some combinations. Therefore, the upper stabilizer content for the pastes to be tested is set to 15 wt.% (the organic content of the paste consists then of 15 wt.% solvent and 15 wt.% stabilizer). This results in a stabilizer-to-nickel mass ratio of 0.21. Since very different stabilizer contents are used in the literature, a second value reduced by a factor of 10 is included in this study which corresponds to a mass fraction of 1.5%. So, both are considered for the paste compositions produced here, implying it is possible for the respective stabilizer–solvent system in terms of miscibility.

The preparation of the nanopastes and the examination of the joining samples reveal some interesting effects of different paste compositions. It has been verified that the addition of a stabilizer, even at a content of only 1.5 wt.%, can be crucial for the manufacturability of a nanopaste with 70 wt.% metal content. This was shown for the pastes utilizing dioctyl ether (DiOE) and paraffin (Pf) as solvents. In the absence of the stabilizer, only crumbly mixtures could be produced, although the total organic content was still 30 wt.%. This demonstrates that a stabilizer helps to disperse the particles more effectively and maintain their separation, even in pastes with a high metal content.

The achievable joining strength is also affected by the stabilizer but in distinct ways. In the solvent systems of DiOE and Pf, a higher content leads to higher strength, at least for OA as a stabilizer. Joining samples with paste Ni (70%)_DiOE_OA (15%) reached 70 MPa. The pastes with DiOE as solvent further provide a direct comparison between OA and stearic acid (SA), which are both fatty acids sharing the same number of carbon atoms (18) in their chain. In comparison to OA, the joining strength for both SA-stabilized pastes (1.5 and 15 wt.% SA) is consistently lower. Joining samples of both pastes reached about 30 MPa, although the stabilizer content was varied by a factor of 10. Even at a low content fraction, SA appears to have a detrimental effect in the joining process, since it is not present in the paste in dissolved form, but as fine precipitations. For handling, pastes containing SA require preheating of the paste itself, the base material, and all equipment to approx. 80 °C to prevent the paste from hardening before application. So, overall, SA is not a suitable stabilizer for the intended nanopastes. Pf-based pastes with OA exhibit behavior similar to those based on DiOE; however, the joining strengths achieved are lower (62.4 MPa vs. 70 MPa). Additionally, during the joining process, evaporating paraffin, as an oily substance, leaves residues in the vacuum chamber and on the components within it. Overall, other solvents appear to be more suitable than paraffin. Pastes containing terpineol as solvent benefit from the addition of a stabilizer, with the composition Ni (70%)_T_KD4 (0.7%) achieving the best overall results. This organic composition is derived from Tseng et al. [39] and was already used in our previous studies, with a stabilizer content by weight of 0.7 being preferred over others tested (1.5 and 3.0). Utilizing PEG 400 as a solvent, nanopastes can be produced without additional stabilizers. The addition of stabilizers either worsened the joining results (1.5 wt.% PVP) or had little effect (1.5 wt.% GA), which confirms statements from the literature that PEG 400 already has a stabilizing effect; at least no significant improvement can be achieved by adding a stabilizer. It is worth mentioning that all organic solvent systems tested (DiOE, Pf, T, and PEG) enable a several months shelf life of the nanopastes. At this point, it is difficult to give the exact reasons for the observations of strength and microstructure depending on the different paste compositions. This would require in-depth analyses of interactions between solvent and stabilizer, volatilization and decomposition behavior, etc., of the chemical systems, which differ significantly from each other from a chemical point of view. So far, this study provides a foundation for which compositions are suitable for Ni nanopastes at all and points out promising chemical systems, which could be a starting point for further investigations.

## 5. Conclusions

In the first part of the experimental work, the influence and the behavior of various chemical substances that can be utilized for preparing a nickel nanopaste with 70 wt.% metal content were examined. To ensure a comprehensive study, water-based nanopastes were also considered. In addition to a simple mixture of nanoparticles and distilled water, compositions with two different stabilizers were included. However, it was found that water is far too volatile in such a system to allow a stable suspension. Moreover, preliminary tests have shown that water-based pastes do not lead to durable joints either.

A broader and more promising field of compositions emerges from organic substances. From a variety of candidates, 13 paste compositions were created and applied to joining samples to evaluate the shear strength and microstructure of the joints. Among all organic solvent–stabilizer systems investigated, nanopastes with dioctyl ether (DiOE) as well as terpineol (T) as solvents exhibit the best properties. Within the DiOE pastes, the addition of oleic acid (OA) leads to favorable results, but overall a nanopaste composition with T and the stabilizer KD4 achieved the highest mechanical durability when used for joining.

## Figures and Tables

**Table 2:** Determined temperatures and holding times (via screening tests) for pre-drying of the pastes, depending on the solvent system.

Solvent Used for Nanopaste	Temperature (°C)	Holding Time (s)
Water	(ultimately not used for nanopastes, see Section 3.1.1)	
Dioctyl ether	180	90
Paraffin (paraffin wax)	140	210
Terpineol, pure	140	90
PEG 400	200	90

**Table 3:** Miscibility of candidates for stabilizers and solvents (red: not possible; yellow: possible with limitation; green: possible).

Solvent Stabilizer	Dioctyl Ether (DiOE)	Paraffin (Pf)	Terpineol (T), Pure	PEG 400 (PEG)
**Oleic acid (OA)** liquid	Easily soluble, 1:1 ratio possible ^1^	Easily soluble when heated, 1:1 ratio possible	(not considered ^2^)	(not considered)
**Stearic acid (SA)** liquid	Soluble only when heated above melting point of SA, when cooled down it separates again		(not considered)	(not considered)
**Glycolic acid (GA)** solid	No visible solubility even at low concentration and heated	Soluble when heated, but GA precipitates form when only slightly cooled	Easily soluble when heated, 1:1 ratio not possible (GA precipitates)	Easily soluble when heated, 1:1 ratio possible
**Polyvinyl-** **pyrrolidon (PVP)** solid	No visible solubility even at low concentration and heated	No visible solubility even at low concentration and heated, PVP forms a crust	Easily soluble when heated, but highly viscous/adhesive at 1:1 ratio	Easily soluble when heated, 1:1 ratio not possible (mushy mixture)
**Hypermer KD4 ** liquid	(not considered)	(not considered)	Easily soluble (only low concentration is intended)	(not considered)

^1^ By weight, this applies to all information in the table. ^2^ Not every possible combination was considered in this study; preference was given to those which were supported by the literature.

**Table 4:** Shear strengths achieved by joining samples of all considered nanopaste compositions, color-coded based on numerical values.

Nanopaste Description	Achieved Shear Strength in MPa		
	Sample #1	Sample #2	Avg. #1–2
Ni (70%)_DiOE_OA (15%)	66.9	73.0	70.0
Ni (70%)_DiOE_OA (1.5%)	52.3	53.4	52.9
Ni (70%)_DiOE_SA (15%)	30.0	29.5	29.7
Ni (70%)_DiOE_SA (1.5%)	47.3	17.0	32.1
Ni (70%)_Pf_OA (15%)	60.7	64.1	62.4
Ni (70%)_Pf_OA (1.5%)	46.6	41.2	43.8
Ni (70%)_T_GA (1.5%)	43.7	48.3	46.0
Ni (70%)_T_PVP (1.5%)	56.9	57.9	57.4
Ni (70%)_T_KD4 (0.7%)	97.6	99.4	98.5
Ni (70%)_T	(0)	63.2	(31.6)
Ni (70%)_PEG_GA (1.5%)	47.3	56.7	52.0
Ni (70%)_PEG_PVP (1.5%)	36.2	33.2	34.7
Ni (70%)_PEG	47.2	54.1	50.7

## Data Availability

The original contributions presented in this study are included in the article. Further inquiries can be directed to the corresponding author.
